# Serum sphingolipidomic analyses reveal an upregulation of C16- ceramide and sphingosine-1-phosphate in hepatocellular carcinoma

**DOI:** 10.18632/oncotarget.7741

**Published:** 2016-02-26

**Authors:** Georgios Grammatikos, Niklas Schoell, Nerea Ferreirós, Dimitra Bon, Eva Herrmann, Harald Farnik, Verena Köberle, Albrecht Piiper, Stefan Zeuzem, Bernd Kronenberger, Oliver Waidmann, Josef Pfeilschifter

**Affiliations:** ^1^ Goethe University Hospital, Pharmazentrum Frankfurt, Institut für Allgemeine Pharmakologie und Toxikologie, Frankfurt am Main, Germany; ^2^ Goethe University Hospital, Medizinische Klinik 1, Frankfurt am Main, Germany; ^3^ Pharmazentrum Frankfurt, Institut für klinische Pharmakologie, Goethe University Hospital, Frankfurt am Main, Germany; ^4^ Goethe University, Department of Medicine, Institute of Biostatistics and Mathematical Modelling, Frankfurt am Main, Germany

**Keywords:** S1P, HCC, biomarker, sphingolipid, dihydroceramide

## Abstract

We have recently shown that major alterations of serum sphingolipid metabolites in chronic liver disease associate significantly with the stage of liver fibrosis in corresponding patients. In the current study we assessed via mass spectrometry serum concentrations of sphingolipid metabolites in a series of 122 patients with hepatocellular carcinoma (HCC) compared to an age- and sex-matched series of 127 patients with cirrhosis. We observed a highly significant upregulation of long and very long chain ceramides (C16-C24) in the serum of patients with HCC as compared to patients with cirrhosis (*P* < 0.001). Accordingly, dihydro-ceramides, synthetic precursors of ceramides and notably sphingosine, sphingosine-1-phosphate (S1P) and sphinganine-1-phosphate (SA1P) were upregulated in patients with HCC (*P* < 0.001). Especially the diagnostic accuracy of C16-ceramide and S1P, assessed by receiver operating curve (ROC) analysis, showed a higher area under the curve (AUC) value as compared to alpha fetoprotein (AFP) (0.999 and 0.985 versus 0.823, *P* < 0.001 respectively). In conclusion, serum levels of sphingolipid metabolites show a significant upregulation in patients with HCC as compared to patients with cirrhosis. Particularly C16-ceramide and S1P may serve as novel diagnostic markers for the identification of HCC in patients with liver diseases. Our data justify further investigations on the role of sphingolipids in HCC.

## INTRODUCTION

Hepatocellular carcinoma (HCC) constitutes a major health burden since it represents the sixth most common cancer and the third leading cause of cancer related mortality worldwide [1]. In most cases HCC occurs within a background of advanced liver disease, namely cirrhosis [2] being the most frequent cause of death in these patients [3]. This urges both basic and translational research approaches to improve current diagnostic tools for early detection and surveillance of HCC. Moreover, lack of treatment options for advanced HCC and shortness of available liver allografts for potentially curable patients render the identification of novel target pathways and biomarkers an eminent research goal.

Targeting sphingolipid (SL) metabolism in cancer has gained significant attention in the last two decades [4, 5] since both proliferation and apoptosis of tumors as well as cancer drug resistance are substantially regulated by SL's [6, 7]. Ceramide (Cer), the bioactive hydrophobic backbone of various complex SL's, has been proposed by many studies as a potent tumor-suppressor molecule activated by common cancer treatment modalities such as chemotherapy and irradiation [4, 5] while abrogation of ceramide generation is observed in tumor tissues resistant to therapy [8, 9]. However, recent studies on Cer's with different chain lengths of the fatty acid bound to the sphingosine backbone have revealed that long-chain (C16-C20) and very-long-chain (C22-C24) Cer's express opposite effects both on cell proliferation [10] as well as on plasma membrane fluidity [11]. On the other hand, sphingosine 1-phosphate (S1P), the functional antagonist of Cer, which is generated by deacylation of Cer to sphingosine (Sph) and its subsequent phosphorylation, constitutes a bioactive SL with a key role in stimulating proliferation and growth of mammalian cells, thus driving and enhancing tumorigenesis [12, 13].

The improved implementation of mass spectrometric methods in the last years has enabled a thorough study of SL metabolite variations in various diseases [14]. Serum or plasma Cer's constitute in the meantime evident disease biomarkers in obesity and diabetes mellitus [15], in acute phase reactions [16], in renal [17] and neurodegenerative [18] disorders, while S1P is implicated as a biomarker in various cancer diseases [19, 20]. Despite the fact that several studies observed an implication of Cer and S1P as major regulators of hepatocellular susceptibility to various stimuli [21, 22] as well as of hepatocarcinogenesis both *in vitro* and in the mouse model [23–26], only limited data are available to date regarding SL's as biomarkers of HCC. In our recently published studies we were the first to report, that long (C16-C20) and very long (C24, C24:1) chain Cer's show significant variations in the serum of patients with non-alcoholic fatty liver disease (NAFLD) and in patients chronically infected with hepatitis C virus (HCV) as compared to healthy individuals [27]. The identified variations of serum SL's were significantly associated with the stage of liver fibrosis and responsiveness to antiviral therapy in chronic HCV infection [28].

Purpose of the current study was to assess serum concentrations of various SL metabolites, especially of Cer's with variable chain lengths and S1P in a well characterized series of patients with cirrhosis, diagnosed with HCC as compared to an age- and sex-matched series of patients with cirrhosis without HCC. We intended to assess the potential diagnostic accuracy of serum SL's in HCC and to evaluate eventual correlations of bioactive SL's to severity and surrogate markers of HCC in order to identify potential novel biomarkers.

## RESULTS

### Patient characteristics

A total of 249 patients with cirrhosis were included in the present study according to the above described criteria. 122 patients were diagnosed with HCC and variations of serum SL's were assessed and compared to an age- and gender-matched series of 127 patients with cirrhosis without HCC. Baseline characteristics of the included patients are shown in Table 1. In order to exclude variations of serum SL's resulting from complications or therapy of cirrhosis and HCC we concentrated our analysis on serum samples obtained at the day of study inclusion in a previous prospective study cohort [29]. Chronic alcohol abuse, chronic HCV and chronic hepatitis B virus (HBV) infection were the major etiologies of chronic liver disease in both patient groups (Table 1).

**Table 1:** Demographic, biochemical and clinical characteristics in patients with liver cirrhosis and in patients with HCC

Parameters	Patients with liver cirrhosis and HCC (*n* = 122)	Patients with liver cirrhosis without HCC (*n* = 127)	*P-value*
**Epidemiologic characteristics**			
Age, years Median (range)	*66 (39–87)*	*62 (39–79)*	0.047
Sex			
Female, *n* = (%)	19 (15.5)	31 (24.4)	0.082
Male, *n* = (%)	103 (84.4)	96 (75.5)	
**Biochemical parameters**			
ALT, IU/l Median (range)	48 (8–309)	27 (6–1268)	< 0.001
AST, IU/l Median (range)	80 (20–535)	47 (15–2823)	< 0.001
γGT, IU/l Median (range)	179 (20–1881)	83 (14–1178)	< 0.001
Bilirubin, mg/dl Median (range)	1.1 (0.2–20)	1.7 (0.3–15)	< 0.001
Creatinine, mg/dl Median (range)	0.89 (0.37–5.2)	1.03 (0.42–5.02)	0.001
ALP, IU/l Median (range)	142 (11–937)	113.5 (34–422)	0.001
Albumin, g/dl Median (range)	3.65 (2.1–5.3)	3.2 (1.7–5.2)	0.001
CRP, mg/dl Median (range)	0.93 (0.03–17.4)	1.19 (0.04–16.84)	0.7
Platelets, /μl Median (range)	152 (22–485)	98 (17–396)	< 0.001
Hemoglobin, mg/dl Median (range)	12.8 (8.2–16.9)	10.4 (7.0–16.0)	< 0.001
INR Median (range)	1.17 (0.87–3.03)	1.34 (0.90–2.76)	< 0.001
AFP, ng/ml Median (range)	24.25 (1.4–65000)	3.5 (1.3–210.3)	< 0.001
**Etiology of liver disease**			
Alcohol abuse, *n* (%)	37 (30.3)	69 (54.3)	0.014
Hepatitis C, *n* (%)	40 (32.7)	34 (26.7)	0.4
Hepatitis B, *n* (%)	23 (18.8)	15 (11.8)	0.1
NASH, *n* (%)	12 (9.8)	2 (1.5)	0.007
Cryptogenic, *n* (%)	7 (5.7)	11 (8.6)	0.4
Other*, *n* (%)	16 (13.1)	11 (8.6)	0.3
**Severity of liver disease**			
MELD Median (range)	10 (6–25)	14 (6–35)	< 0.001
Child-Pugh-stage			
A, *n* (%)	66 (54.0)	24 (18.8)	< 0.001
B, *n* (%)	34 (27.8)	67 (52.7)	0.009
C, *n* (%)	13 (10.6)	35 (27.5)	0.005
**Severity of HCC**			
BCLC-stage			
A, *n* (%)	26 (21.3)	-	-
B, *n* (%)	45 (36.8)		-
C, *n* (%)	32 (26.2)	-	-
D, *n* (%)	14 (11.4)	-	-

Median with range or number of patients with percent in brackets. Abbreviations: HCC: hepatocellular carcinoma, ALT: alanine aminotransferase, AST: aspartate aminotransferase, γGT: gamma-glutamyl-transferase, ALP: alkaline phosphatase, CRP: C-reactive protein, INR: international normalized ratio, AFP: alpha-fetoprotein, NASH: non-alcoholic steatohepatitis, MELD: Model of End stage Liver Disease, BCLC; Barcelona Clinic Liver Cancer.

Missing data: ALT levels were missing in 2 HCC patients, AST levels were missing in 2 HCC patients, γGT levels were missing in 2 HCC patients, Bilirubin levels were missing in 1 HCC patient, ALP levels were missing in 3 HCC patients and in 1 patient with liver cirrhosis, Albumin levels were missing in 4 HCC patients and in 2 patients with liver cirrhosis, CRP levels were missing in 31 HCC patients and in 3 patients with liver cirrhosis, Hemoglobin levels were missing in 1 HCC patient, AFP levels were missing in 14 HCC patients and in 32 patients with liver cirrhosis.

### Serum long and very long chain (dihydro-)ceramides as well as SA1P and S1P are upregulated in HCC

**Figure 1:**
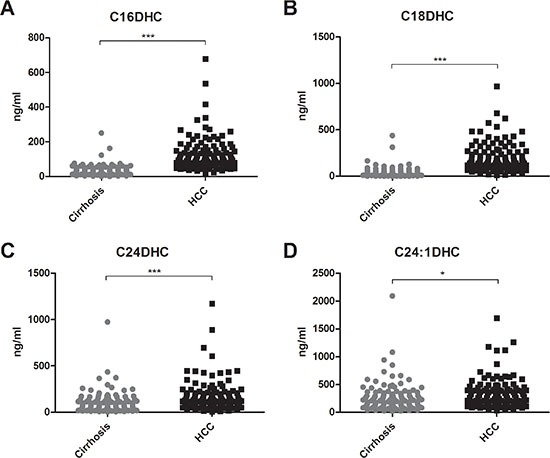
Serum dihydroceramides are upregulated in HCC patients Both long chain DHC's (C16DHC and C18DHC) as well as very long chain DHC's (C24DHC and C24:1DHC) show significantly higher concentrations in the serum of HCC patients as compared to patients with liver cirrhosis (*P* < 0.001 for C16DHC, C18DHC, C24DHC and *P* < 0.05 for C24:1DHC). DHC: dihydroceramide.

**Figure 2:**
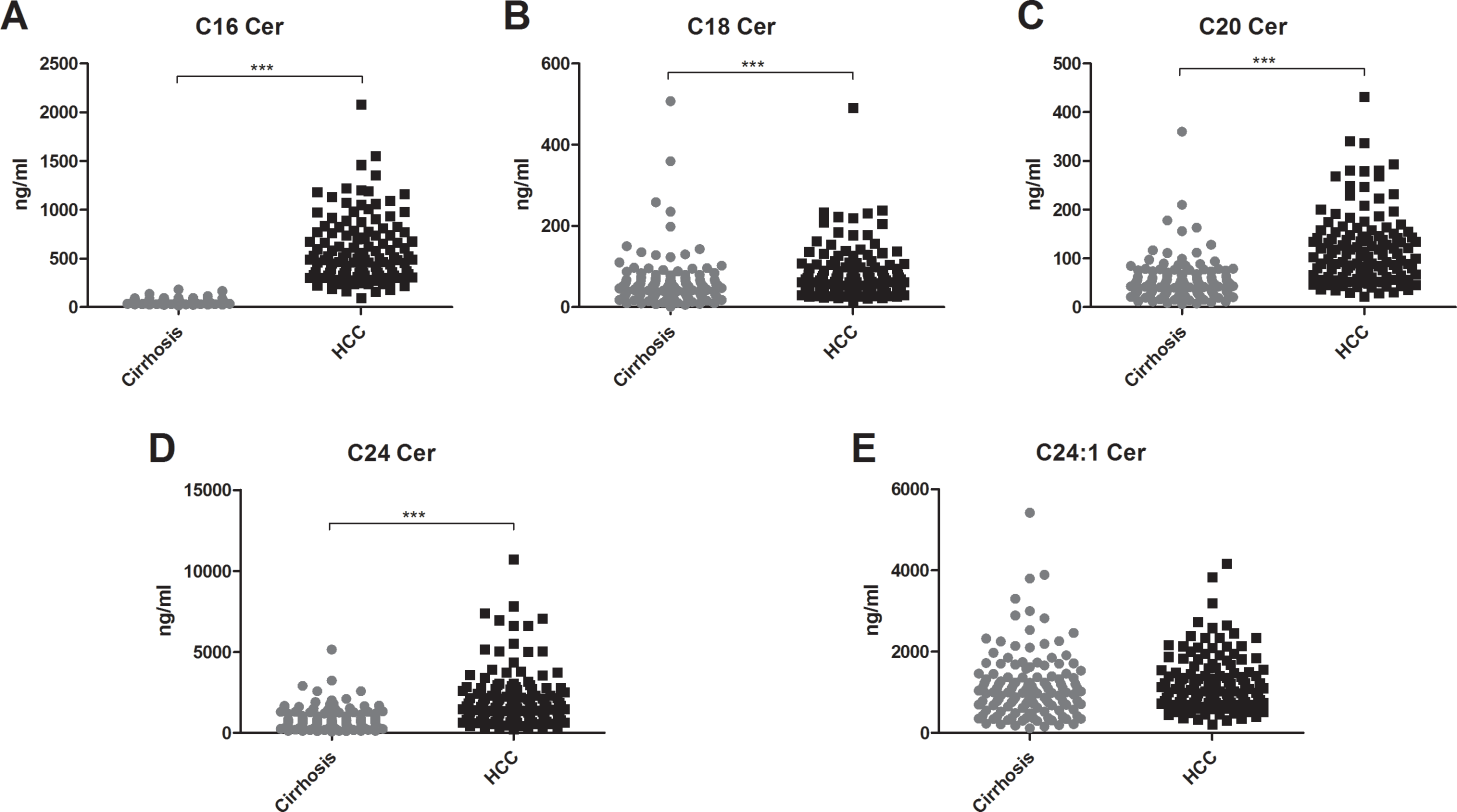
Ceramides accumulate in the serum of patients with HCC Except for the unsaturated derivative of C24Cer, C24:1Cer (2E), all serum Cer's assessed were upregulated in the serum of HCC patients as compared to patients with liver cirrhosis (*P* < 0.001). Cer: ceramide.

**Figure 3:**
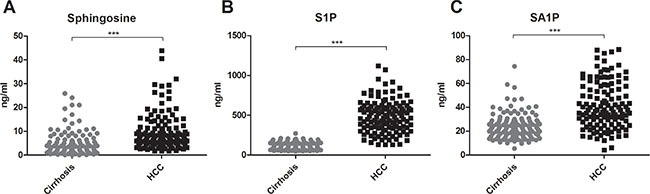
Sphingosine, S1P and SA1P in HCC patients Serum sphingosine, S1P and SA1P are highly elevated in HCC patients as compared to patients with liver cirrhosis (*P* < 0.001). S1P: sphingosine 1-phosphate, SA1P: sphinganine 1-phosphate.

Serum concentrations of long (C16-C20) and very long (≥ C24) chain Cer's as well as of their synthetic precursors, dihydroceramides (DHC's), were assessed in patients with HCC and in patients with cirrhosis. We observed an accumulation of both DHC's (Figure 1) and of Cer's (Figure 2) in HCC as compared to cirrhosis. Except from C24:1Cer and C24:1DHC, the respective unsaturated derivatives of C24Cer and C24DHC, all Cer's and DHC's analyzed showed a highly significant accumulation in HCC as compared to cirrhosis (Figures 1A, 1B, 1C and 2A, 2B, 2C and 2D, *P* < 0.001). Sphingosine, the bioactive amino-alcohol backbone of SL's, was upregulated in the serum of HCC patients as well (Figure 3A, *P* < 0.001). Both its phosphate derivative S1P as well as the phosphate derivative of sphinganine, sphinganine 1-phosphate (SA1P) showed a significant accumulation in HCC as compared to cirrhosis (Figure 3B, 3C, *P* < 0.001, respectively). Further, we evaluated possible associations between serum SL concentrations and severity of HCC according to the BCLC stage. Thereby, no significant variations in serum SL's were identified among the different BCLC stages (Supplementary Figures S1, S2 and S3).

### Correlations of sphingolipid parameters with biochemical and surrogate markers of HCC

Since SL parameters showed a significant accumulation in the serum of HCC patients we further used Spearman rank correlations to identify potential relationships to demographic characteristics and biochemical parameters in these patients. We identified significant correlations of various DHC's and Cer's with markers of hepatocellular injury, while none of the parameters assessed, correlated significantly with the age of the included patients (Table 2). Regarding the severity of liver disease, solely C16DHC and C16Cer showed a positive correlation with the MELD score (Table 2, *P* < 0.05 and *P* < 0.001 respectively) whereas levels of C20Cer, C24Cer, S1P and SA1P were inverse proportional to the MELD score (Table 2, *P* < 0.01, *P* < 0.001, *P* < 0.001 and *P* < 0.05 respectively). Interestingly, despite the fact that all of the SL parameters assessed showed an accumulation in the serum of HCC patients (Figures 1, 2 and 3), only a part of them associated with alpha fetoprotein (AFP), the only broadly available HCC biomarker in the clinical setting to date. Particularly, C16DHC, C18DHC, C24:1DHC, C16Cer, C18Cer and sphingosine correlated significantly with the levels of AFP (Table 2, *P* < 0.01, *P* < 0.001, *P* < 0.01, *P* < 0.001, *P* < 0.05 and *P* < 0.01 respectively).

**Table 2:** Correlation of serum SL's with age, MELD score and biochemical parameters in HCC patients

SL	Age	AST	ALT	γGT	AFP	CRP	Hb	MELD
C16DHC	0.065	0.409***	0.317***	0.406***	0.294**	0.155	−0.21*	0.208*
C18DHC	0.096	0.321***	0.235*	0.428***	0.331***	0.182	−0.151	0.096
C24DHC	0.054	0.076	0.197*	0.223*	0.119	−0.211*	0.104	−0.123
C24:1DHC	0.021	0.297**	0.313***	0.381***	0.3**	**−**0.044	−0.036	0.109
C16Cer	0.044	0.348***	0.273**	0.406***	0.347***	0.162	−0.154	0.257***
C18Cer	0.134	0.18	0.168	0.465***	0.224*	0.317**	−0.023	−0.138
C20Cer	0.126	0.176	0.243**	0.436***	0.16	0.199	0.028	−0.237**
C24Cer	0.118	−0.169	0.042	0.149	−0.012	**−**0.191	0.259**	−0.444***
C24:1Cer	0.104	0.16	0.249**	0.417***	0.201*	0.144	0.065	−0.177
Sphingosine	−0.106	0.19*	0.197*	0.263**	0.307**	0.084	0.113	0.091
S1P	−0.151	−0.134	0.003	0.146	0.135	**−**0.041	0.320***	−0.375***
SA1P	−0.125	−0.146	−0.117	0.093	0.068	**−**0.124	0.169	−0.22*

Correlation is evaluated by Spearman's correlation coefficient rho (r). Abbreviations: AST: aspartate aminotransferase, ALT: alanine aminotransferase, γGT: gamma glutamyl transferase, AFP: alpha fetoprotein, CRP: C reactive protein, Hb: hemoglobin, MELD: Model of End stage Liver Disease, DHC: dihydroceramide, Cer: ceramide, S1P: sphingosine 1-phosphate, SA1P: spinganine 1-phosphate. Significant correlations are shown in bold and are indicated in the corresponding Figures: “*”= *p* < 0.05, “**”= *p* < 0.01, “***”= *p* < 0.001.

Missing data: AST levels were missing in 2 patients, ALT levels were missing in 2 patients, γGT levels were missing in 2 patients, AFP levels were missing in 14 patients, SA1P levels were missing in 3 patients, S1P levels were missing in 3 patients, Sphingosine levels were missing in 3 patients, C24:1DHC levels were missing in 2 patients, C24DHC levels were missing in 2 patients, C18DHC levels were missing in 2 patients, C16DHC levels were missing in 2 patients, C24:1Cer levels were missing in 2 patients, C20Cer levels were missing in 2 patients, C18Cer levels were missing in 2 patients, C16Cer levels were missing in 2 patients, C24Cer levels were missing in 2 patients.

### Serum C16Cer and S1P show a high diagnostic accuracy in differentiating patients with HCC from cirrhotic patients

**Figure 4:**
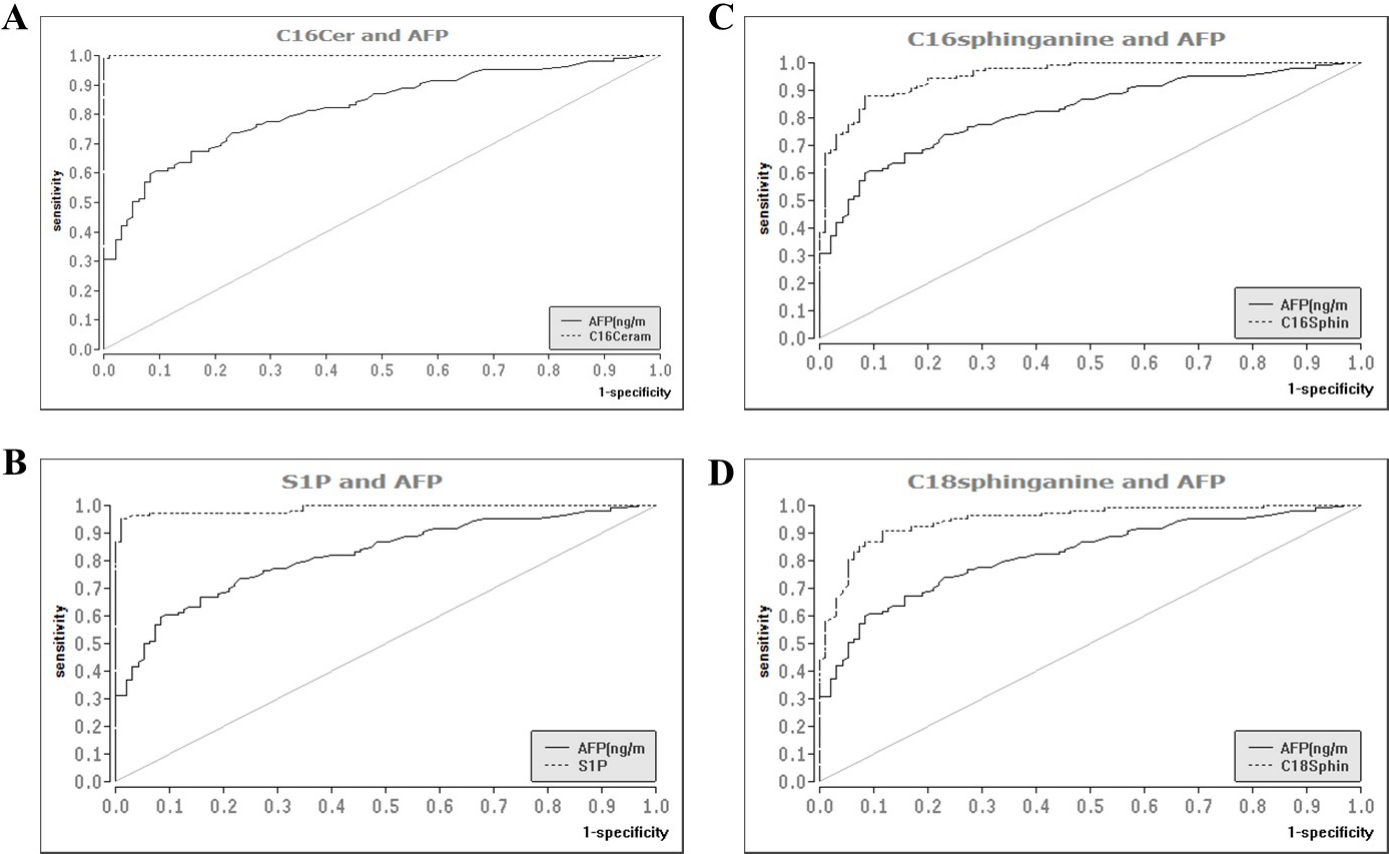
Diagnostic performance of serum sphingolipids as compared to AFP in the differentiation of HCC from liver cirrhosis ROC analysis identified serum SL parameters with a superior diagnostic accuracy as compared to AFP, the only widely available serologic marker of HCC. ROC: receiver operating curve, SL: sphingolipid, AFP: alpha fetoprotein, AUC: area under the curve, HCC: hepatocellular carcinoma.

Since nearly all of the SL's analyzed associated significantly in univariate analysis with the presence of HCC, we intended to conduct a multivariate analysis to evaluate if SL parameters constitute independent diagnostic predictors of HCC. However, a multivariable logistic regression analysis was computationally not feasible since some of the SL parameters, particularly C16Cer and S1P, provided a nearly complete diagnostic separation of HCC patients from patients with liver cirrhosis. We further assessed the diagnostic performance of serum SL's by ROC analysis with the estimation of correspondent AUC. AUC values, cut-off parameter concentrations and estimation of the wrong classification rate (WCR) are listed in Supplementary Table 1. C16Cer and S1P showed the highest AUC values (0.999 and 0.985, *P* < 0.001 respectively) and consequently also the lowest WCR-values (1.2% and 4.2% respectively), being thus more accurate than AFP (WCR of 24.1%) in the differentiation of HCC from cirrhotic patients. Moreover, C16DHC and C18DHC appeared also with a high diagnostic accuracy, since their AUC values (for both parameters 0.932, *P* < 0.001) were significantly higher than that of AFP (0.823, *P* < 0.001). Corresponding ROC graphs of serum SL's as compared to AFP are illustrated in Figure 4. Finally we evaluated a diagnostic algorithm in the primary diagnosis of HCC and especially BCLC A HCC according to the above mentioned cut-off concentrations of C16Cer and S1P (Supplementary Figure 4). AFP, C16Cer and S1P levels were accessible in 106 out of 122 HCC patients and in 22 out of 26 BCLC A HCC patients. Only 33 HCC patients (30.5%) could be diagnostically identified by AFP levels, while C16Cer and S1P were able to identify additional 72 (67.5%) and 70 (64.8%) HCC patients respectively (Supplementary Figure 4A, 4C). In patients with BCLC A HCC the diagnostic benefit from C16Cer and S1P was even more substantial since additional 20 patients with AFP levels < 200 ng/ml (88.4%) could be identified by C16Cer and S1P (Supplementary Figure 4B, 4D).

## DISCUSSION

Several studies on molecular level and in a mouse model have linked SL metabolism with the pathogenesis of HCC [23–26]. Yet, sphingolipidomics in HCC have been only sparsely studied so far and offered limited data regarding alterations of serum SL parameters in patients with HCC. In our recent studies we identified significant variations of serum SL's in chronic liver disease [27] with marked associations of specific SL's to the stage of liver fibrosis and outcome of interferon-based antiviral therapy in HCV patients [28]. Thus, purpose of the current study was to evaluate the serum SL profile of patients with HCC and verify possible variations of SL parameters with potential diagnostic and experimental value both for the clinical setting as well as for novel research approaches.

Data from a recently published review on the progressive implementation of metabolomics for the identification of HCC biomarkers highlighted parameters and derivatives of the bile acid, phospholipid and sphingolipid metabolism as potential HCC biomarkers [30]. Studies on serum SL's in human subjects offered conflicting data regarding alterations of S1P in patients with HCC as compared to cirrhotic patients also lacking a simultaneous evaluation of further parameters of the SL metabolism [31, 32]. In our current analysis we observed a significant elevation of S1P serum concentrations in HCC with a marked diagnostic specificity and sensitivity compared to further biochemical parameters and AFP (Table 2, Figures 3B and 4B). In line with our current observations, previous *in vitro* studies revealed that overexpression of sphingosine kinase 1 (SK1), which catalyzes the phosphorylation of sphingosine to S1P, stimulated migration and invasiveness of HCC cell lines with SK1-mRNA levels being upregulated in HCC biopsies [23]. Inhibition of SK1 by cinobufotalin, a bufadienolide derivative, suppressed proliferation of HCC *in vitro* [33] and inhibition of SK2, a further SK isoform, expressed a potent antitumor activity in HCC cell lines [34]. However, the relevance of elevated serum SA1P levels in HCC patients remains enigmatic since *in vitro* studies investigating the role of SA1P in hepatocarcinogenesis are lacking so far.

We further observed a considerable upregulation of serum concentrations of Cer's with distinct acyl chain lengths (Figure 2) and of their respective synthetic precursors, DHC's (Figure 1) in the serum of HCC patients. While the role of Cer in tumorigenesis is well studied and extensively reviewed [5, 35], upcoming evidence from various studies points to chain length-specific effects of Cer's [36, 37]. Particularly, long chain Cer's (C16-C20) show anti-proliferative effects, while very long chain Cer's (≥ C24) appear as pro-proliferative [37]. Ablation of Cer-generating enzymes, as for instance Cer-synthase 2, which predominantly catalyzes the production of very long chain Cer's, has been shown to deregulate substantially liver homeostasis and to activate hepatocarcinogenesis in the mouse model [38]. On the other hand, depletion of the pro-oncogenic chaperone gp96 caused a paradoxical enhancement of liver tumorigenesis due to accumulation of long chain Cer's [25]. In our current study both long chain and very long chain Cer's showed an accumulation in the serum of patients with HCC (Figure 2). Especially, C16Cer correlated with markers of hepatocellular injury and AFP (Table 2) and also showed the highest diagnostic specificity and sensitivity in the differentiation of HCC from cirrhotic patients among all SL parameters tested (Supplementary Table 1, Figure 4A). C16Cer levels showed a positive trend to correlate with higher BCLC stages, though no significant association of serum C16Cer and BCLC stage was identified in our study (Supplementary Figure 1C). Although beyond the primary scope of our study we may assume that elevated serum levels of long chain Cer's may stimulate hepatocarcinogenesis in patients with HCC in accord with the above mentioned observations from basic research studies [25, 37]. Further studies are needed in order to elucidate the exact role of long and very long chain Cer's in HCC.

DHC's constitute synthetic precursors of Cer's via the *de novo* anabolic pathway of SL metabolism. Here we identified a highly significant upregulation of DHC's in patients with HCC (Figure 1). DHC's correlated positively with transaminases and AFP (Table 2) and showed a marked discriminative potential in the serologic diagnosis of HCC (Supplementary Table 1 and Figure 4C, 4D). From a mechanistic point of view DHC's are potent regulators of autophagy [37], a physiologic process that facilitates the turnover of proteins and organelles thus playing a major role in determining the cell fate in pathophysiologic processes of cellular stress or tumorigenesis [39]. In liver pathophysiology the role of autophagy remains controversial with most of the studies observing a suppression of hepatocarcinogenesis when autophagy is impaired [40–42]. Since DHC's are activating autophagy [43] and an accumulation of DHC's is observed in the serum of HCC patients in our study, we may hypothesize that elevated levels of DHC's may stimulate hepatocarcinogenesis by induction of autophagic pathways in these patients. However, the exact role of autophagy and DHC's in hepatocarcinogenesis should be deciphered in further studies.

Although offering very promising results, our study had some limitations. Our current retrospective clinical association study cannot prove a causal relationship between SL metabolism and HCC. Nevertheless, our observations indicate a link between SL's and HCC and thus a novel framework for the design of future *in vitro* studies and eventually prospective clinical trials. Despite our effort to provide a comprehensive analysis of the sphingolipidomic profile of HCC, data on further SL metabolites such as sphinganine or sphingomyelin were not available in our study. Moreover, the observed non-significant trends between serum levels of SL's and BCLC stage indicate that a higher number of patients in each BCLC stage should be included in future studies in order to evaluate with accuracy possible associations of serum SL's with the stage of HCC. Additionally, since S1P levels show a significant variation between serum and plasma samples [14], our results are restricted to the analysis of serum and not plasma. Plasma samples were unfortunately not available in the current study. In spite of these limitations, our results offer a significant advance over previous studies given that they highlight a marked association between serum SL concentrations and occurrence of HCC in a well characterized series of patients with cirrhosis. Interestingly, C16Cer and S1P prevailed significantly in their diagnostic accuracy over AFP, the main broadly available serologic biomarker of HCC which lacks diagnostic sensitivity and specificity.

In conclusion, our study identifies significant alterations of SL parameters in the serum of HCC patients and emphasizes the potential of sphingolipidomics in order to query novel biomarkers within the serologic signature of HCC. Certainly, further *in vitro* and *in vivo* studies are necessary to shed light into the pathophysiologic links between SL metabolism and HCC. Prospective interventional clinical trials affecting SL regulating enzymes may be justified in patients with HCC.

## PATIENTS AND METHODS

In the current study, we evaluated the association between serum SLs and the presence and stage of HCC in a series of 249 patients. Overall, 122 patients with cirrhosis and HCC and 127 patients with cirrhosis without HCC were included in our analysis. The study was performed in accord with the Declaration of Helsinki and was approved by the local ethics committee. All patients had signed a written informed consent before study inclusion.

### HCC patients

Serum samples of 122 patients with HCC who were followed between February 2009 and April 2013 at the Department of Internal Medicine 1 of the Johann Wolfgang Goethe University Hospital Frankfurt, Germany were routinely stored and used for the present study. Patients were included in a previously published prospective cohort [29]. The diagnosis of HCC was made according to the EASL practice guidelines [44] by histopathology or by dynamic imaging with characteristic hypervascularity in the arterial phase and washout in the portal venous phase. Exclusion criteria were an age below 18, history of cancer other than HCC in the last five years, history of solid organ transplantation and local or systemic treatment for HCC within the last 28 days. The diagnostic potential of SL parameters was assessed at the day of study inclusion. The Barcelona Clinic Liver Cancer (BCLC) stage, model of end stage liver disease (MELD) score and Child Pugh stage were assessed by clinical examination, laboratory parameters and the results of abdominal ultrasound examination, computed tomography or magnetic resonance imaging at the time of inclusion in the study.

### Patients with cirrhosis

An age- and gender-matched control group of 127 patients with comparable liver function was derived from a previously published cohort of patients with cirrhosis who were treated in our department and initially participated from March 2009 until June 2011 in a prospective cohort study [45]. From this cohort, patients were selected for the present study as follows: For each HCC patient included in the present study, 1 patient with cirrhosis was randomly matched according to age and gender. Before matching, both patient cohorts were stratified in groups according to age (18–29, 30–39, 40–49, 50–59, and > 60). Within these groups, patients were randomly matched for gender. Randomization was performed based on a numerical order of a random identification number, which had been assigned in the original prospective study in which all patients with cirrhosis had been included [45]. Inclusion criteria were cirrhosis, proven by histopathological examination of liver biopsy material or explicit morphological criteria of cirrhosis in ultrasound, computed tomography or magnetic resonance imaging and an age ≥ 18 years. Exclusion criteria were a history of malignant disease within the last five years and former solid organ or bone marrow transplantation.

### Determination of SL concentrations by high-performance liquid chromatography-tandem mass spectrometry

Quantification of serum SLs was performed by high-performance liquid chromatography-tandem mass spectrometry (LC-MS/MS), as previously described [28]. Collected blood samples from patients were centrifuged for 10 minutes at 3000 rpm. Serum was collected under sterile conditions and was aliquoted in cryo-tubes, which were subsequently stored at −80°C. For quantitation of SLs, 20 μL of serum were extracted with methanol/chloroform/HCl (15:83:2). Afterwards, amounts of C16:0Cer, C18:0Cer, C20:0Cer, C24:1Cer, C24:0Cer, C16:0dhCer, C18:0dhCer, C24:0dhCer, C24:1dhCer, and the internal standard (C17:0Cer) and sphingosine, S1P and SA1P, and the internal standards (sphingosine-D7, sphinganine-D7 and sphingosine 1-phosphate-D7) were analyzed by LC-MS/MS. All serum samples were stored at −80°C until assayed. A Luna C18 column (150 mm × 2 mm ID, 5 μm particle size, 100 Å pore size; Phenomenex, Aschaffenburg, Germany) was used for chromatographic separation. The HPLC mobile phases consisted of water-:formic acid (100:0.1, v/v) (A) and acetonitrile-:tetrahydrofuran-:formic acid (50:50:0.1, v/v/v) (B). For separation, a gradient program was used at a flow rate of 0.3 ml/min. The initial buffer composition 60% (A)/40% (B) was hold for 0.6 min and then in 3.9 min linearly changed to 0% (A)/100% (B) and hold for 6.5 min. Subsequently the composition was linearly changed within 0.5 min to 60% (A)/40% (B) and then held for another 4.5 min. The running time for every sample (injection volume: 15 μl for Cer and dhCer determination and 10 μl for the other sphingolipids) was 16 min. MS/MS analyses were performed on a API4000 (triple quadrupole mass spectrometer) equipped with an APCI (Atmospheric Pressure Chemical Ionization) ion source (AB Sciex, Darmstadt, Germany) for Cer and dhCer determination, and with an ESI (Electrospray Ionization) ion source for sphingosine, sphinganine and their 1-phosphate derivatives determination. The analysis was done in Multiple Reaction Monitoring (MRM) mode. For every analyte two transitions were recorded: one for quantification and another one for qualification, to exclude false positive results, with a dwell time of 50 ms. For analysis and quantification the Analyst Software 1.5 (AB Sciex, Darmstadt, Germany) was used and the peak area of the analyte was corrected by the peak area of the internal standard. Linearity of the calibration curve was proven for C16:0Cer, C24:0Cer, C16:0dhCer, C24:1dhCer, C24:0dhCer from 0.6 to 1.000 ng/ml, for C18:0Cer from 0.18 to 300 ng/ml, for C20:0Cer, C24:1Cer from 0.24 to 400 ng/ml and for C18:0dhCer from 0.3 to 500 ng/ml. For sphingosine, S1P and SA1P the calibration curve ranged from 0.15 to 250 ng/ml. The coefficient of correlation was at least 0.99. Variations in accuracy were less than 15% over the whole range of calibration.

### Statistical analysis

Statistical analysis for the scatter and box plots presented was performed with GraphPad Prism for Windows (v5.01; GraphPad Software Inc., San Diego, CA). Further statistical calculations were performed by using BiAS software for Windows (version 10.11; Epsilon-Verlag, Darmstadt, Germany). Statistical comparisons for continuous variables were carried out using the nonparametric Mann-Whitney's U and Kruskal-Wallis' tests to determine differences among patient groups. Dichotomic variables were assessed by means of contingency tables (Mantel-Haenszel's test), as appropriate. The data are expressed as means ± standard error, unless otherwise specified. The level of significance was set at a = 0.05, representing the 95% confidence interval (CI). Statistically significant differences are indicated in the corresponding figures (*P < 0.05; **P < 0.01; ***P < 0.001). The correlation coefficient rho was calculated by using the Spearman correlation provided in BiAS software. Receiver operating characteristic (ROC) curves and area under the curve (AUC) values were calculated by the BiAS software as well. *P* values are not corrected for multiple testing.

## SUPPLEMENTARY MATERIALS FIGURES AND TABLE



## Abbreviations

HCC: hepatocellular carcinom
SL: sphingolipid
Cer: ceramide
S1P: sphingosine 1-phosphate
Sph: sphingosine
NAFLD: non-alcoholic fatty liver disease
HCV: hepatitis C virus
BCLC: Barcelona Clinic Liver Cancer
MELD: model of end stage liver disease
LC-MS/MS: liquid chromatography/tandem mass spectrometry
ROC: receiver operating curve
AUC: area under the curve
APCI: Atmospheric Pressure Chemical Ionization
ESI: electrospray ionization
MRM: multiple reaction monitoring
HBV: hepatitis B virus
DHC: dihydroceramide
SA1P: sphinganine 1-phosphate
AFP: alpha fetoprotein
WCR: wrong classification rate
SK1: sphingosine kinase 1
TNFα: tumor necrosis factor alpha.
