# Supplementary material for: Serum sphingolipidomic analyses reveal an upregulation of C16- ceramide and sphingosine-1-phosphate in hepatocellular carcinoma
Source: Oncotarget. 2016 Feb 26;7(14):18095–105. doi: 10.18632/oncotarget.7741 (PMC4951274; doi:10.18632/oncotarget.7741)
Supplement: Supplementary file 1 [file oncotarget-07-18095-s001.pdf]

Serum sphingolipidomic analyses reveal an upregulation of C16-ceramide and sphingosine-1-phosphate in hepatocellular carcinoma

Supplementary Materials

Supplementary Table S1: AUC values of liver enzymes, MELD score and further biochemical and SL parameters in the prediction of HCC in patients with liver cirrhosis

Parameters	AUC ± SD	95% CI	Cut-off value	WCR	*P*-value
ALT	0.742 ± 0.031	0.680–0.804	35.5 IU/l	29.1%	< 0.001
AST	0.732 ± 0.032	0.669–0.795	63.5 IU/l	30.9%	< 0.001
γGT	0.699 ± 0.033	0.633–0.764	105.5 IU/l	33.8%	< 0.001
CRP	0.514 ± 0.041	0.433–0.594	0.445 mg/l	45.2%	0.7
AFP	0.823 ± 0.028	0.766–0.879	7.45 ng/ml	24.1%	< 0.001
Hb	0.730 ± 0.031	0.668–0.793	11.35 g/dl	29.7%	< 0.001
MELD	0.736 ± 0.031	0.675–0.797	10.5	30.8%	< 0.001
C16DHC	0.932 ± 0.015	0.901–0.963	55.2 ng/ml	13.4%	< 0.001
C18DHC	0.932 ± 0.016	0.900–0.965	64.2 ng/ml	11.7%	< 0.001
C24DHC	0.684 ± 0.033	0.618–0.749	65.1 ng/ml	36.3%	< 0.001
C24:1DHC	0.604 ± 0.035	0.533–0.674	301 ng/ml	40.5%	0.004
C16Cer	0.999 ± 0.000	0.998–1.000	147.5 ng/ml	1.2%	< 0.001
C18Cer	0.665 ± 0.034	0.597–0.732	67.7 ng/ml	36.3%	< 0.001
C20Cer	0.837 ± 0.025	0.788–0.886	78.6 ng/ml	22.1%	< 0.001
C24Cer	0.826 ± 0.025	0.775–0.877	953 ng/ml	23.8%	< 0.001
C24:1Cer	0.572 ± 0.036	0.501–0.643	431 ng/ml	42.6%	0.048
Sphingosine	0.759 ± 0.031	0.697–0.821	4.83 ng/ml	27.8%	< 0.001
S1P	0.985 ± 0.005	0.974–0.997	215 ng/ml	4.2%	< 0.001
SA1P	0.790 ± 0.029	0.733–0.848	30.8 ng/ml	23.8%	< 0.001

The diagnostic performance of serum SL's, assessed by receiver operating characteristic (ROC) curve analysis with the estimation of correspondent areas under the curve (AUC). Abbreviations: *CI: confidence interval, SD: standard deviation, WCR: wrong classification rate, ALT: alanine aminotransferase, AST: aspartate aminotransferase, γGT: gamma-glutamyl-transferase, AFP: alpha fetoprotein, CRP: C reactive protein, Hb: hemoglobin, MELD: Model of End stage Liver Disease, DHC: dihydroceramide, Cer: ceramide, S1P: sphingosine 1-phosphate, SA1P: spinganine 1-phosphate.*
Missing data: AST levels were missing in 2 patients, ALT levels were missing in 2 patients, γGT levels were missing in 2 patients, AFP levels were missing in 14 patients, SA1P levels were missing in 3 patients, S1P levels were missing in 3 patients, Sphingosine levels were missing in 3 patients, C24:1DHC levels were missing in 2 patients, C24DHC levels were missing in 2 patients, C18DHC levels were missing in 2 patients,C16DHC levels were missing in 2 patients, C24:1Cer levels were missing in 2 patients, C20Cer levels were missing in 2 patients, C18Cer levels were missing in 2 patients, C16Cer levels were missing in 2 patients, C24Cer levels were missing in 2 patients.

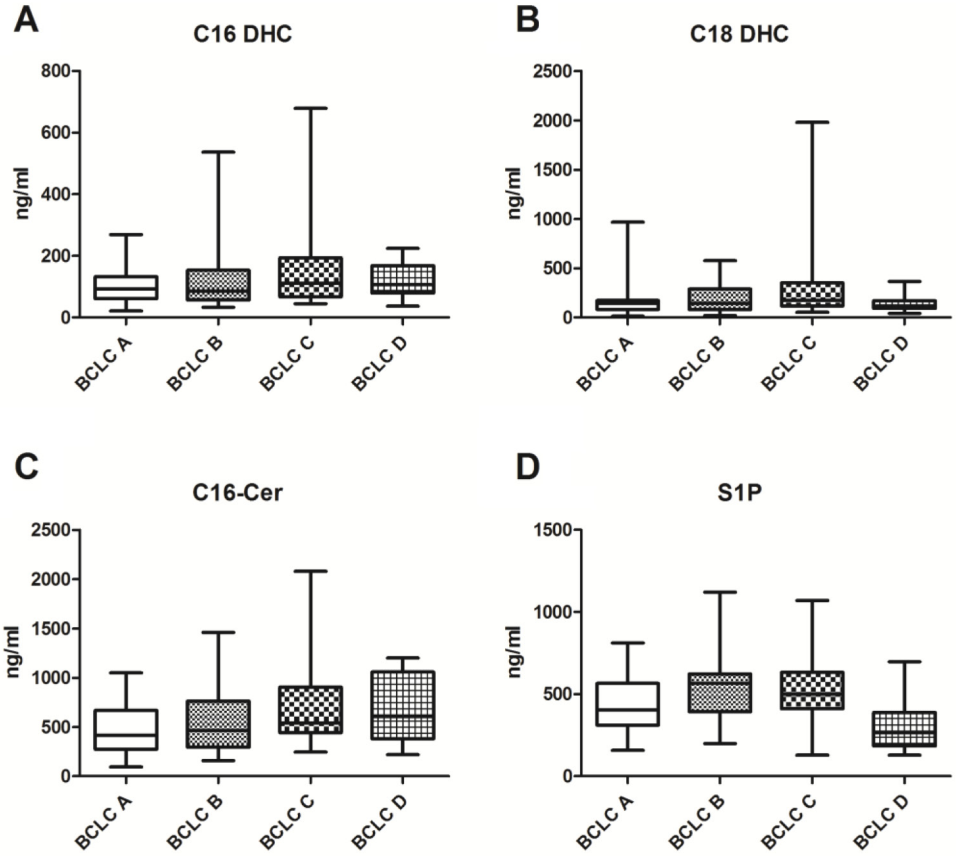

Supplementary Figure S1: Serum SL parameters and BCLC stage. No significant variations between serum concentrations of various SL parameters and BCLC stage of HCC were observed in our study. SL: sphingolipid, BCLC: Barcelona Clinic Liver Cancer, HCC: hepatocellular carcinoma, Cer: ceramide, DHC: dihydroceramide, S1P: sphingosine 1-phosphate.

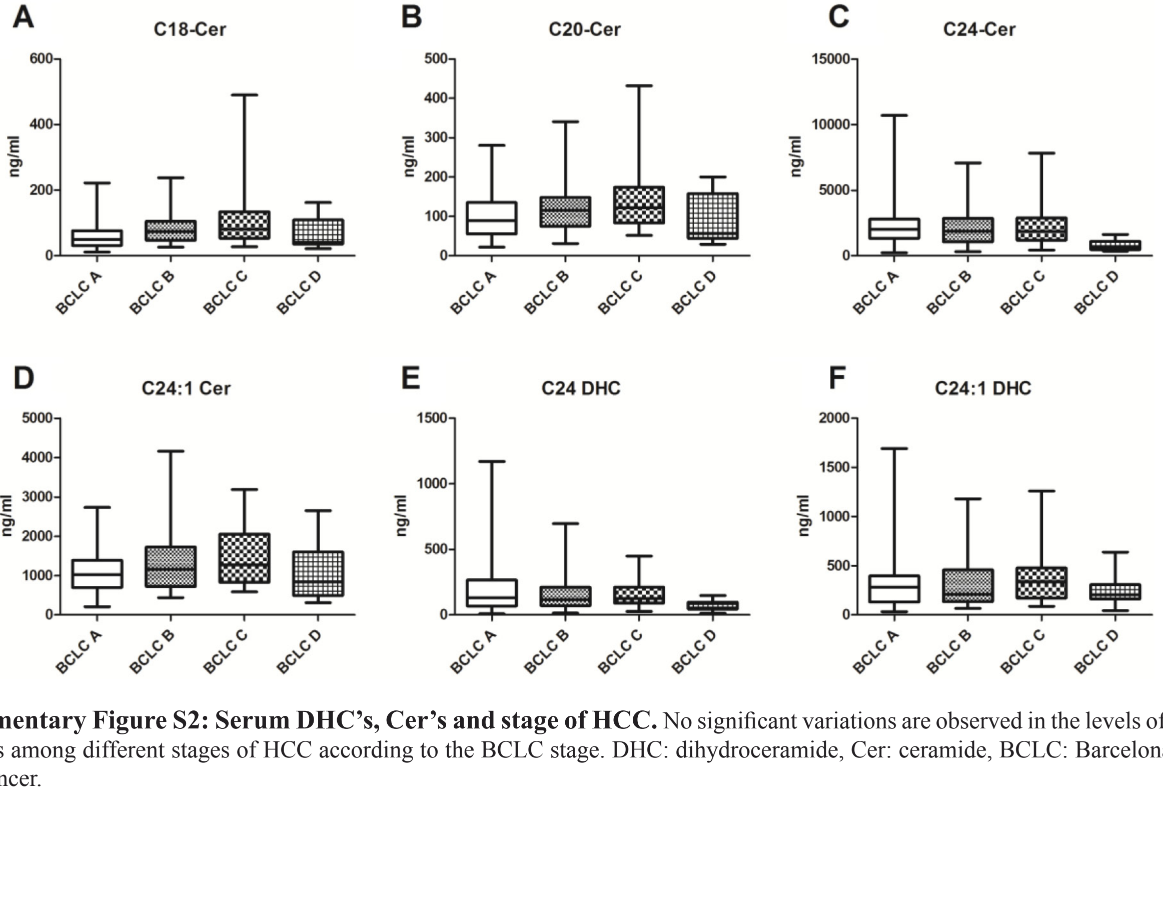

Supplementary Figure S2: Serum DHC's, Cer's and stage of HCC. No significant variations are observed in the levels of DHC's and Cer's among different stages of HCC according to the BCLC stage. DHC: dihydroceramide, Cer: ceramide, BCLC: Barcelona Clinic Liver Cancer.

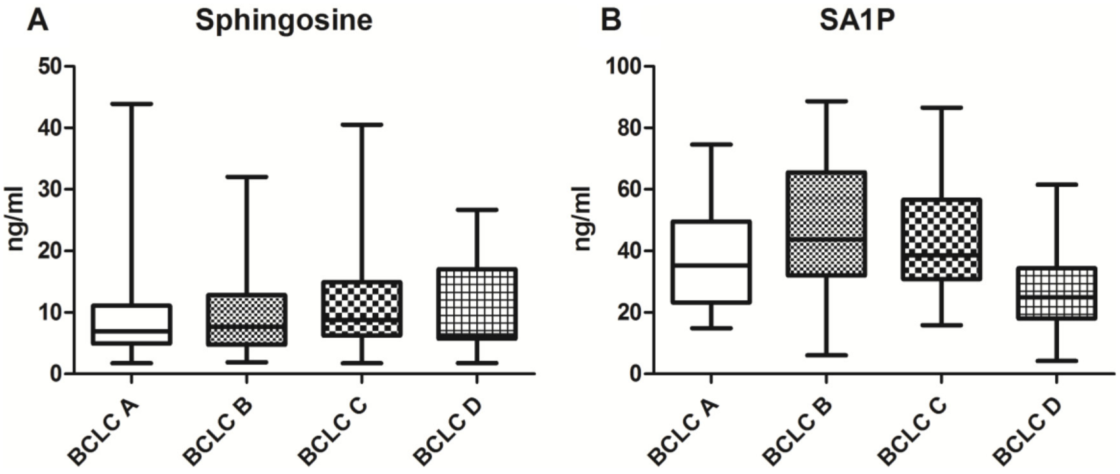

Supplementary Figure S3: Serum sphingosine and SA1P and stage of HCC. Despite the observed positive trend between serum levels of sphingosine, SA1P and the BCLC stage no significant variations were observed. SA1P: sphinganine 1-phosphate, BCLC: Barcelona Clinic Liver Cancer.

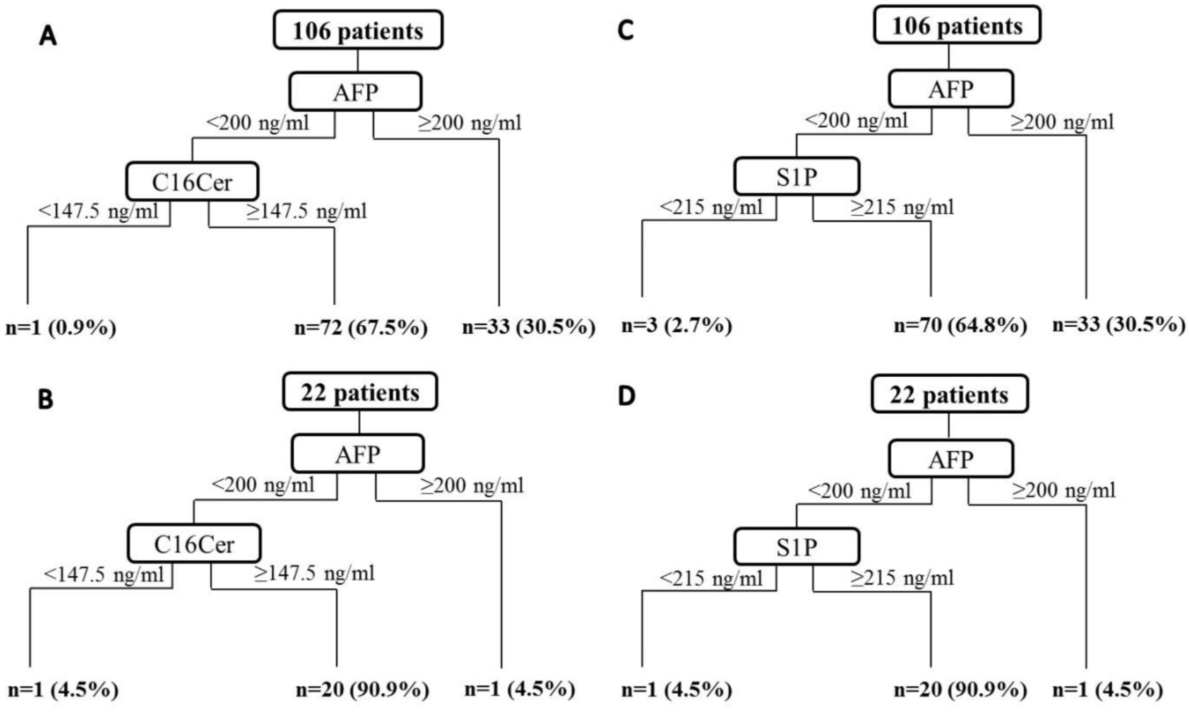

Supplementary Figure S4: Diagnostic algorithm of non-invasive diagnosis of HCC by C16Cer or S1P. Implementation of the diagnostic algorithm of non-invasive HCC diagnosis according to AFP, C16Cer or S1P in all HCC patients (**A, C**) and in patients with an early stage BCLC A HCC (**B, D**). 106 out of 122 patients with HCC and 22 out of 26 BCLC A HCC patients were included in the algorithm where both AFP and C16Cer or S1P were available. HCC: hepatocellular carcinoma, Cer: ceramide, S1P: sphingosine 1-phosphate, BCLC: Barcelona Clinic Liver Cancer.
